# Recurrent pemphigoid gestationis in the setting of first-trimester spontaneous abortions

**DOI:** 10.1016/j.jdcr.2024.10.003

**Published:** 2024-10-28

**Authors:** Madeline Ngo, Rohit Gupta, Edward Hadeler, Yevgeniya Rainwater

**Affiliations:** a University of Texas Southwestern Medical School, Dallas, Texas; b Department of Dermatology, University of Texas Southwestern Medical Center, Dallas, Texas; c Dermatopathology Division, Sonic USA/Propath, Dallas, Texas

**Keywords:** first trimester, pemphigoid gestationis, spontaneous abortion, vesiculobullous disorders

## Introduction

Pemphigoid gestationis (PG) is a rare, autoimmune subepidermal blistering disease, occurring in approximately 1 in 50,000 pregnancies.^1^ In this disease, abnormal expression of major histocompatibility complex class II antigens in the placenta stimulates maternal antibody production, which bind to the BP180/230 antigens in the placenta and cross react with BP180/230 antigens in the basement membrane of the skin. This results in activation of the inflammatory complement cascade, causing subepidermal blister formation.^2^ Clinically, PG presents as pruritic urticarial papules and plaques followed by the development of clustered vesicles and tense bullae on the abdomen with distinctive periumbilical involvement, also involving extremities, and, rarely, mucous membranes.^3^ PG usually occurs during the second or third trimester of pregnancy and may flare postpartum. Recurrence in successive pregnancies is frequent.^4^ While PG is associated with a higher risk of premature delivery and infants that are small-for-gestational-age, there is no well-established elevated risk of miscarriages in the setting of PG.^5^ Herein, we report a case of recurrent PG in the setting of spontaneous abortions in the first trimester.

## Case report

**Fig 1:**
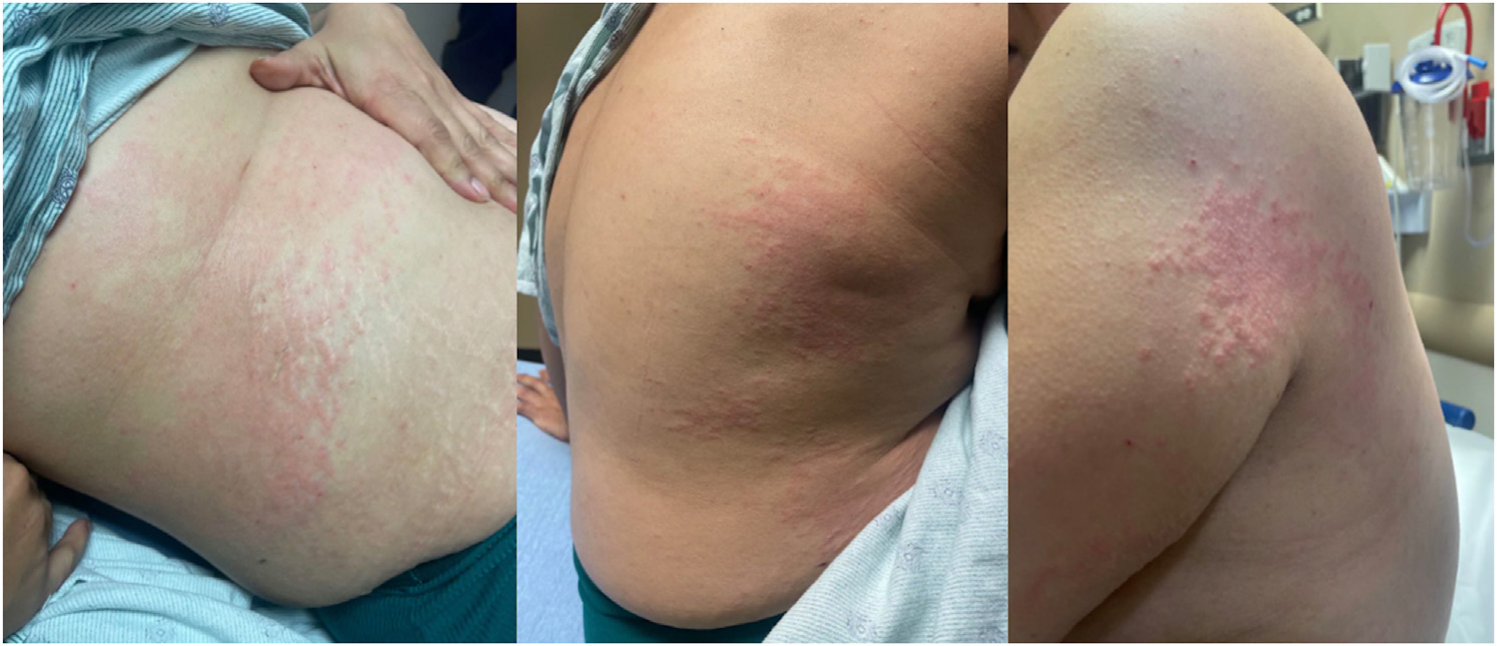
Erythematous, edematous papules, and plaques on the trunk and proximal extremities.

**Fig 2:**
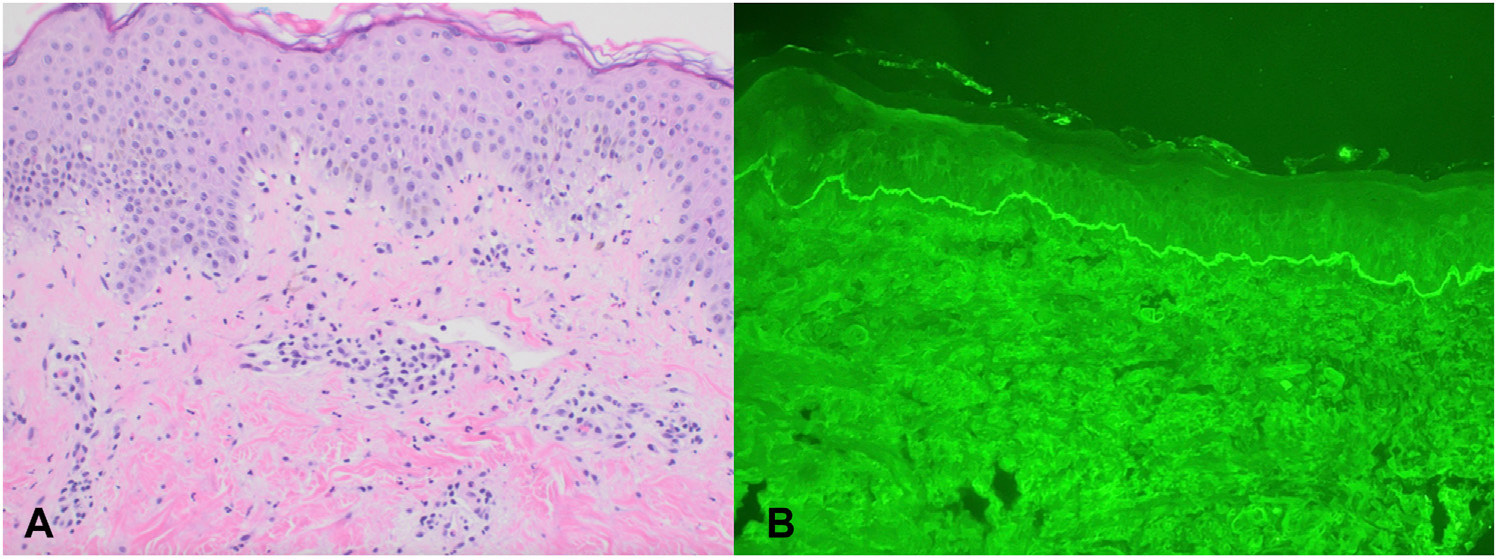
**A,** Histopathological examination demonstrated mild spongiosis with a perivascular and interstitial inflammatory cell infiltrate comprising eosinophils and neutrophils with eosinophils preset at dermal-epidermal junction (DEJ). **B,** Direct immunofluorescence with linear deposition of immunoglobulin G (IgG) along DEJ.

A 44-year-old G9P6A2 female with a remote history of “bullous pemphigoid” presented to the emergency department (ED) with heavy vaginal bleeding and intensely pruritic eruption involving her trunk and extremities. Physical examination revealed scattered, excoriated, erythematous, edematous papules, and plaques involving proximal extremities, thighs, and abdomen with periumbilical involvement (Fig 1). Patient was also found to have a positive pregnancy test with ultrasound confirming a 12-week gestation. Given concern for a pregnancy-related dermatosis, skin biopsies for hematoxylin and eosin and direct immunofluorescence (DIF) were performed demonstrating an urticarial pattern of inflammation without vesiculation or subepidermal blister formation (Fig 2). DIF studies showed a linear deposition of immunoglobulin G and complement 3 along the basement membrane. Indirect immunofluorescence revealed positive anti-BP180 antibodies (27 U/mL). Taken together, these findings were most concerning for PG. Concurrently, obstetrics was consulted for heavy vaginal bleeding, and subsequent evaluation confirmed that the patient underwent a complete spontaneous abortion. The patient was treated with a short prednisone taper with a prompt resolution of the eruption and pruritus.

Notably, 14-years prior to this presentation, the patient was seen in our ED with a similar pruritic eruption. A skin biopsy was performed at that time demonstrating a slight spongiotic dermatitis with eosinophils. Similarly, DIF revealed a linear deposition of immunoglobulin G and complement 3 along the dermal-epidermal junction. Given negative pregnancy test at that time, a working diagnosis of BP was made. The patient rapidly improved on a combination of systemic steroids, dapsone, and doxycycline. Upon further questioning, however, the patient mentioned that she experienced a miscarriage within a month of developing this pruritic eruption. Medical record review confirmed that 3 weeks before her initial presentation to the ED the patient underwent a complete spontaneous abortion at 7 weeks gestation. In view of this additional history, her current presentation is best regarded as a recurrence of PG. Interestingly, between her initial and current presentation, the patient had 3, healthy, term pregnancies with no recurrence of pruritus or rash.

## Discussion

PG is generally regarded as a disease of a second and third trimester of pregnancy, unlikely to be associated with significant maternal and fetal morbidity and mortality aside from prematurity and intrauterine growth restriction. Herein we present a unique case of a recurrent PG developing in the first trimester of pregnancy in association with a concurrent pregnancy loss. While onset in the first trimester has been reported in 16% to 31% of patients with PG,^6^^,^^7^ association between PG and pregnancy loss is not well-recognized. A 2020 systematic review of 109 articles including 140 PG patients, demonstrated a total of 7 spontaneous abortions and 1 stillbirth.^8^ Another study of 87 patients with PG (278 total pregnancies) demonstrated a much higher rate of spontaneous abortions at 16%.^4^ Importantly, of 44 spontaneous abortions, 34 (77.3%) occurred in the first trimester.^4^ Our case report adds to this growing body of evidence suggesting that PG may carry a higher risk of pregnancy loss than previously thought, especially when it develops in the first trimester of pregnancy. It has been suggested that placental insufficiency caused by antibody deposition within the chorionic villi may play a pathogenic role in poor fetal outcomes. It is plausible that early onset of PG in the first trimester of pregnancy leads to a more severe placental insufficiency and more serious fetal complications including spontaneous abortion. In any case, it is important to consider additional causes for first trimester spontaneous abortions, including the role of advanced maternal age in our case. Future studies are needed to clarify the association between PG and first trimester spontaneous abortions among other physiologic and pathologic factors.

Another unique aspect of this patient’s presentation is 3 uninvolved, term pregnancies intervening the initial and current presentations of PG. PG is typically associated with a 50% rate of recurrence in subsequent pregnancies, and uninvolved or “skipped” pregnancies are rare.^6^ In a retrospective study of 87 patients with PG, “skipped” pregnancies were reported in only 8% of cases.^4^ While the reasons for “skipped” pregnancies remain enigmatic, the authors of this study conclude that neither compatibility of HLA-DR loci between mother and fetus nor a change in paternity appear to be contributing factors.^4^

In summary, this case report of a recurrent first trimester PG flares with associated repeat pregnancy losses underscores the importance of expanding our view of this disease beyond the scope of a second/third trimester pregnancy-related dermatosis with minimal fetal mortality. This report and review of literature demonstrates that when developing in the first trimester of pregnancy, PG may be associated with a significant risk of pregnancy loss. Therefore, it is important for dermatologists to promptly evaluate and diagnose pregnant patients with pruritic eruptions as well as facilitate referral to maternal fetal medicine service for close monitoring and high-risk pregnancy management.

## Conflicts of interest

None disclosed.
